# Post-mortem serum concentrations of GFAP correlate with agony time but do not indicate a primary cerebral cause of death

**DOI:** 10.1371/journal.pone.0205323

**Published:** 2018-10-10

**Authors:** Benedict Breitling, Robert Brunkhorst, Marcel Verhoff, Christian Foerch

**Affiliations:** 1 Department of Neurology, Goethe-University, Frankfurt am Main, Germany; 2 Department of Forensic Medicine, Goethe-University, Frankfurt am Main, Germany; Massachusetts General Hospital/Harvard Medical School, UNITED STATES

## Abstract

**Background and purpose:**

The astroglial protein GFAP is a blood biomarker indicative of intracerebral hemorrhage in patients with acute stroke. Due to its brain specificity and the necessity of brain damage for its detectability in blood, we hypothesized that GFAP could be an interesting marker in cases with primary cerebral cause of death, e.g., traumatic brain injury.

**Methods:**

All corpses scheduled for an autopsy in the Frankfurt Department of Forensic medicine within a 15-month period were included in the study. Cases with a known history of brain disease in the 3 months before death were excluded. During autopsy, blood was collected and GFAP serum levels were determined using a commercially available ELISA. The autopsy protocols were reviewed for the presence of a primary cerebral or a primary non-cerebral cause of death. Agony time was also determined.

**Results:**

A total of 129 autopsy cases were included. GFAP concentrations did not differ between cerebral (median 0.96 μg/l, IQR 5.03) and non-cerebral causes of death (1.21 μg/l, 3.58). GFAP levels were found to be unaffected by hemolysis or post-mortem interval. GFAP levels were found to be increased in cases with prolonged agony times (median 1.76 μg/l [IQR 4.70]) compared to short (0.58 μg/l [0.58]; p<0.001) and ultra-short agony times (0.21 μg/l [0.12]; p = 0.002).

**Conclusion:**

Post-mortem GFAP serum concentrations correlate with agony time and might therefore be useful for the evaluation of the severity of brain damage in prolonged death. Elevated GFAP serum levels do not indicate a primary cerebral cause of death.

## Introduction

Glial fibrillary acidic protein (GFAP) is an intermediate filament protein produced by astrocytes in order to maintain cell stability and shape.[1, 2] It is abundantly present in the brain but was also identified in other tissues (such as bone marrow) in low quantity.[1–3] Healthy individuals typically do not reveal detectable GFAP levels in their blood stream.[4, 5, 6] However, substantial damage to the CNS (i.e., damage associated with astroglial necrosis and cellular disintegration) causes rapid GFAP release into CSF which trespass into the blood stream through an affected blood-brain barrier.[7] In particular, intracerebral hemorrhage [8, 9] and traumatic brain injury[10, 11] are associated with strong GFAP elevations in serum. Ischemic and hypoxic conditions also cause GFAP release.[12, 13] The amount of brain tissue destruction directly correlates with GFAP serum concentrations.[7, 9, 10] On the other side, GFAP is not increased in mild cerebral injury not affecting the structural integrity of astrocytes.[5] Moreover, transient cerebral ischemia does not cause higher GFAP concentrations, as long as ischemic cell death and necrosis do not occur.[14] Previous studies showed that GFAP can be detected in post mortem brain specimen, both from humans and rodents. [15, 16] Here, GFAP expression is particularly present in areas of reactive astrogliosis after traumatic brain injury and stroke. [11, 15].

In view of these findings, GFAP blood levels could be an interesting marker in forensic medicine. We hypothesized that post-mortem GFAP levels will be high if a primary cerebral event (either “intrinsic,” such as intracerebral or subarachnoid hemorrhage, or “extrinsic,” such as traumatic brain injury) is the cause of death. However, until now, no data on the GFAP release kinetics into the blood in the peri-mortal phase is available, and it is unclear whether brain death *per se* (regardless of the underlying reason) provokes a GFAP elevation or not. A prospective study was set up to investigate these issues.

## Materials and methods

### Study design

This prospective study was undertaken between January 2015 and April 2016 at the Department of Neurology and the Department of Forensic Medicine, Hospital of the Goethe University, Frankfurt am Main, Germany. All procedures performed in studies involving human participants were in accordance with the ethical standards of the institutional ethic committee of the University Frankfurt, Germany (internal reference number: 216/13) and with the 1964 Helsinki declaration and its later amendments or comparable ethical standards. Need for consent was waived by the ethics committee.

All corpses scheduled for a forensic autopsy in the institute were considered for study inclusion. Exclusion criteria were (I) age of the deceased individual <18 years, (II) known brain disease in the medical history within 3 months before death, and (III) severely decomposed bodies.

The autopsy protocols were reviewed and the following parameters were prospectively documented: age, sex, medical history, immediate hospitalization before death, mean time span from time-of-death to blood collection, resuscitation prior to death, and autopsy results. For each case, the cause of death was determined on the basis of the autopsy examination, including macro-morphological, histological, and toxicological analyses.

The primary endpoint of the study was the presence of a primary cerebral cause of death (vs. a primary non-cerebral cause of death). A primary cerebral cause of death was determined in case of brain pathologies (e.g. hemorrhage, traumatic brain injury, hypoxic brain damage) that were considered to be the main cause of death in the final autopsy report. In a secondary analysis we enriched the primary cerebral death group with all cases revealing macroscopic changes in brain integrity (such as cerebral edema and cerebral hemorrhage) and all cases in which the brain was exposed to hypoxia or ischemia (i.e., respiratory cause of death, exsanguination, multi-organ-failure, resuscitation).

Agony time was stratified into three subgroups[17, 18]: (I) ultra-short (a period of only a few seconds) was assumed in cases with high-speed-trauma; (II) short (a period of a few seconds up to a few minutes) was assumed in cases where drowning, mechanical asphyxia, and shot-, stab- or cut-wounds led to death; (III) prolonged (a period lasting a few minutes up to hours) was assumed in cases with intoxication or resuscitation.

### Blood sampling and GFAP measurement

During autopsy, blood was drawn aseptically from the right cardiac chambers using syringes. Blood was collected in standard forensic and toxicological glass tubes. Samples were centrifuged, and serum was stored in Eppendorf tubes at -80°C. Later on, samples were shipped on dry ice.

GFAP serum levels were measured at Randox (North Ireland) with standardized ELISA kits (for technical details: http://www.randoxresearch.com/elisa#gfap). The calibration range was reported to be 0-107ng/ml, and the sensitivity was 0.29ng/ml. The intra-assay and inter-assay precision was <8%. All persons involved in the measuring procedure of the samples were fully blinded to the clinical data. Prior to GFAP measurement, the degree of hemolysis was assessed visually with a semi-quantitative rating scale ranging from 1 to 5 (1 = no hemolysis, 5 = severe hemolysis). In addition, hemolytic indices (a standard method for hemolysis interference evaluation) were determined on a clinical analyzer (for technical details: https://www.randox.com/clinical-chemistry-analysers/).

### Statistical analysis

Statistical analysis was performed using the SPSS statistical package Version 24 (Statistical Package for the Social Sciences). Parametric and non-parametric data was determined by using the Kolmogorov Smirnov Test. Comparisons between two groups were performed using the t-test (tT) for parametric data, the Mann Whitney U-test (MWU) for non-parametric data, and the chi-square (CQ) test for categorical variables. Comparisons between three groups were made using the non-parametric Kruskal-Wallis-Test (KW). Post-hoc analyses were performed by means of the Mann-Whitney U test (MWU). Correlation analyses were performed by means of the non-parametric Spearman rank (S) test.

## Results

The study comprised 129 autopsy cases. The mean age of the cohort was 58±19 years; 44 cases (34%) were females. The mean time span between death and blood draw was 110±67 h. A primary cerebral cause of death was identified in 18 cases (14%). Hereof, 12 cases had traumatic brain injury (4 car crash, 3 fall, 2 blunt force trauma, 1 gunshot, 1 train crash), 3 had subarachnoid hemorrhage (3 ruptured aneurysm), 2 had intracerebral hemorrhage, and 1 had a subdural hematoma. Within the group of cases with a non-neurological cause of death (n = 111), 40 cases died from a primary cardiac event, 28 died from a primary respiratory cause, 25 died of intoxication, 11 died of exsanguination and 8 died of multi-organ failure. Forty-eight cases (37%) were resuscitated immediately before death. Baseline variables of the study cohort are depicted in Table 1.

**Table 1:** Characteristics of the study population.

	all	cerebral cause of death	non cerebral cause of death	p
n (%)	129 (100)	18 (14.0)	111 (86.0)	
Mean age, years (SD)	58.2 (19.2)	61.4 (24.0)	57.7 (18.4)	0.443
Men, n (%)	85 (65.9)	12 (66.7)	73 (65.8)	0.94
Mean BMI (SD)	26.74 (6.2)	26.2 (4.7)	26.8 (6.4)	0.927
Post mortem interval, h (SD)	110.4 (66.8)	112.9 (44.4)	110.0 (69.9)	0.338
Medical history / pre-existing conditions, n (%)				
a. cerebrovascular disease (Intracerebral hemorrhage or ischemic stroke)	6 (4.7)	0	6 (5.4)	0.312
b. cardiovascular disease	43 (33.3)	6 (33.3)	37 (33.3)	0.999
c. pulmonary disease	21 (16.3)	3 (16.7)	18 (16.2)	0.962
d. diabetes mellitus	15 (11.6)	2 (11.1)	13 (11.7)	0.941
e. cancer	11 (8.5)	2 (11.1)	9 (8.1)	0.672
f. neurological disorders	23 (17.8)	4 (22.2)	19 (17.1)	0.6
g. drug abuse	35 (27.1)	4 (22.2)	31 (27.9)	0.614
h. psychiatric disorders	20 (15.5)	1 (5.6)	19 (17.1)	0.209
i. others	43 (33.3)	4 (22.2)	39 (35.1)	0.281
Resuscitation, n (%)	48 (37.2)	5 (27.8)	43 (38.7)	0.372
Resuscitation time, min (SD)*	73.5 (50.8)	57.5 (46.0)	74.8 (51.9)	0.594
Immediate hospitilization before death, n (%)	43 (33.3)	10 (55.6)	33 (29.7)	0.031
Autopsy results				
a. cerebral edema, n (%)	72 (55.8)	12 (66.7)	60 (54.1)	0.318
b. macroscopic cerebral hemorrhage, n (%)	19 (14.7)	15 (83.3)	4 (3.6)	<0.001
c. macroscopic changes in brain integrity (stroke, cancer), n (%)	31 (24.0)	13 (72.2)	18 (16.2)	<0.001
d. pulmonary edema, n (%)	63 (48.8)	7 (38.9)	56 (50.5)	0.327
e. coronary heart disease and vascular sclerosis, n (%)	53 (41.1)	5 (27.8)	48 (43.2)	0.216
f. myocardial disease (infarction or myopathy), n (%)	52 (40.3)	5 (27.8)	47 (42.3)	0.243
g. vascular rupture, n (%)	17 (13.2)	3 (16.7)	14 (12.6)	0.637
h. cancer, n (%)	9 (7.0)	1 (5.6)	8 (7.2)	0.799
i. fractures, n (%)	28 (21.7)	10 (55.6)	18 (16.2)	<0.001
j. brain weight, g (SD)	1358.8 (186.5)	1349.3 (249.3)	1363.2 (176.1)	0.992
k. degree of hemolysis, 1 to 5 (SD)	3.0 (1.2)	2.7 (1.6)	3.1 (1.15)	0.378
l. others, n (%)	60 (46.5)	6 (33.3)	54 (48.6)	0.201

* if known

The average brain weight was found to be in a range between 700g to 1900g (mean 1359±187 g). Post-mortem examination indicated that 72 cases (56%) had cerebral edema, 19 (15%) had macroscopic cerebral hemorrhage, and 31 (24%) had macroscopic changes in brain integrity (Table 1).

We found a positive correlation between the degree of hemolysis and the time span from death to blood draw (semi-quantitative determination: p<0.001, r = 0.391; hemolytic indices: p<0.001, r = 0.476(S)). In contrast, no correlation was found between hemolysis and GFAP serum levels (semi-quantitative determination: p = 0.264, r = -0.114; hemolytic indices: p = 0.245, r = -0.142(S)). Time to blood draw and GFAP levels did not correlate (p = 0.554, r = 0.05 (S)). GFAP values were found unaffected by age and were evenly distributed between females and males (Table 1).

GFAP concentrations were obtained in 125 out of 129 samples (four samples gave errors). Median GFAP value for the entire cohort was 1.16 μg/l (IQR 3.87, minimum 0.00, maximum 76.25). There was no significant difference in GFAP levels between primary cerebral (median 0.96 μg/l, IQR 5.03, minimum 0.12, maximum 9.91) and non-cerebral causes of death (1.21 μg/l, 3.58, 0.00, 76.25; p = 0.749(MWU)). Similarly, GFAP levels did not differ between cases with brain injury (including the cases with a primary cerebral cause of death, cases with macroscopic brain damage and cases in which the brain was exposed to hypoxia or ischemia) and cases without brain injury (1.02 μg/l [3.24, 0.00, 76.25]; n = 91 vs. 1.39 μg/l [4.94, 0.05, 38.0]; n = 34, p = 0.967 (MWU)). In addition, we examined GFAP concentrations separately in cases with cerebral hemorrhage (n = 19, median 1.15μg/l, IQR 8.93, minimum 0.12, maximum 37.65) and those without (n = 102, median 1.13μg/l, IQR 3.31, minimum 0.00, maximum 76.25). We were not able to identify differences between the two groups (p = 0.324(MWU)). GFAP concentrations in the 43 cases with documented resuscitation did not differ significantly from those without (p = 0.131(MWU)).

**Fig 1:**
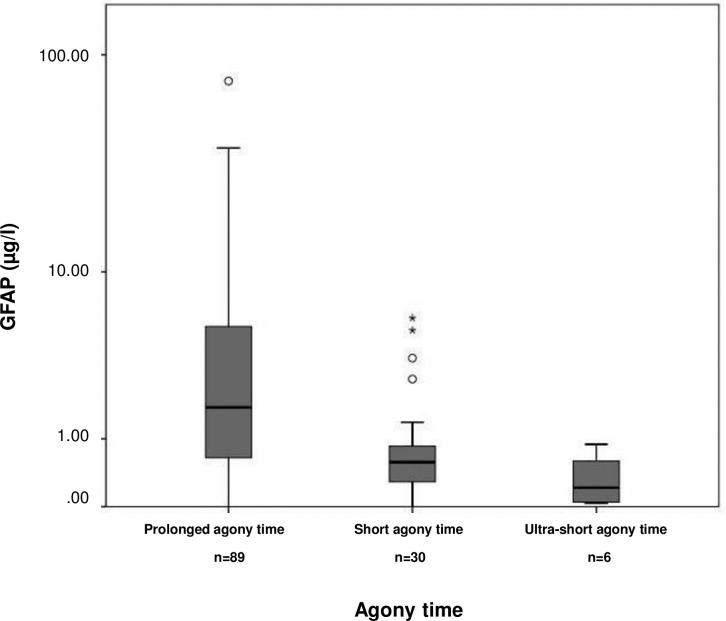
Post-mortem GFAP serum concentrations of the study population stratified according to agony time. The boundaries of the box indicate the 25^th^ and 75^th^ percentile, respectively. The whiskers indicate the 10^th^ and 90^th^ percentile, respectively. Outliers (between 1.5 and 3 times the interquartile range) are marked with circles. Extreme values (> 3 times the interquartile range) are marked with asterisks. The y-axis is on a logarithmic scale. GFAP concentrations: ultra-short agony times (median 0.21 μg/l [IQR 0.12, minimum 0.03, maximum 0.89]; n = 6), short agony times (0.58 μg/l [0.58, 0.00, 5.87]; n = 30), and prolonged agony times (1.76 μg/l [4.68, 0.00, 76.25], n = 89; Kruskal Wallis p<0.001; post-hoc ultra-short versus prolonged agony times p = 0.002, short versus prolonged agony times p<0.001).

**Fig 2:**
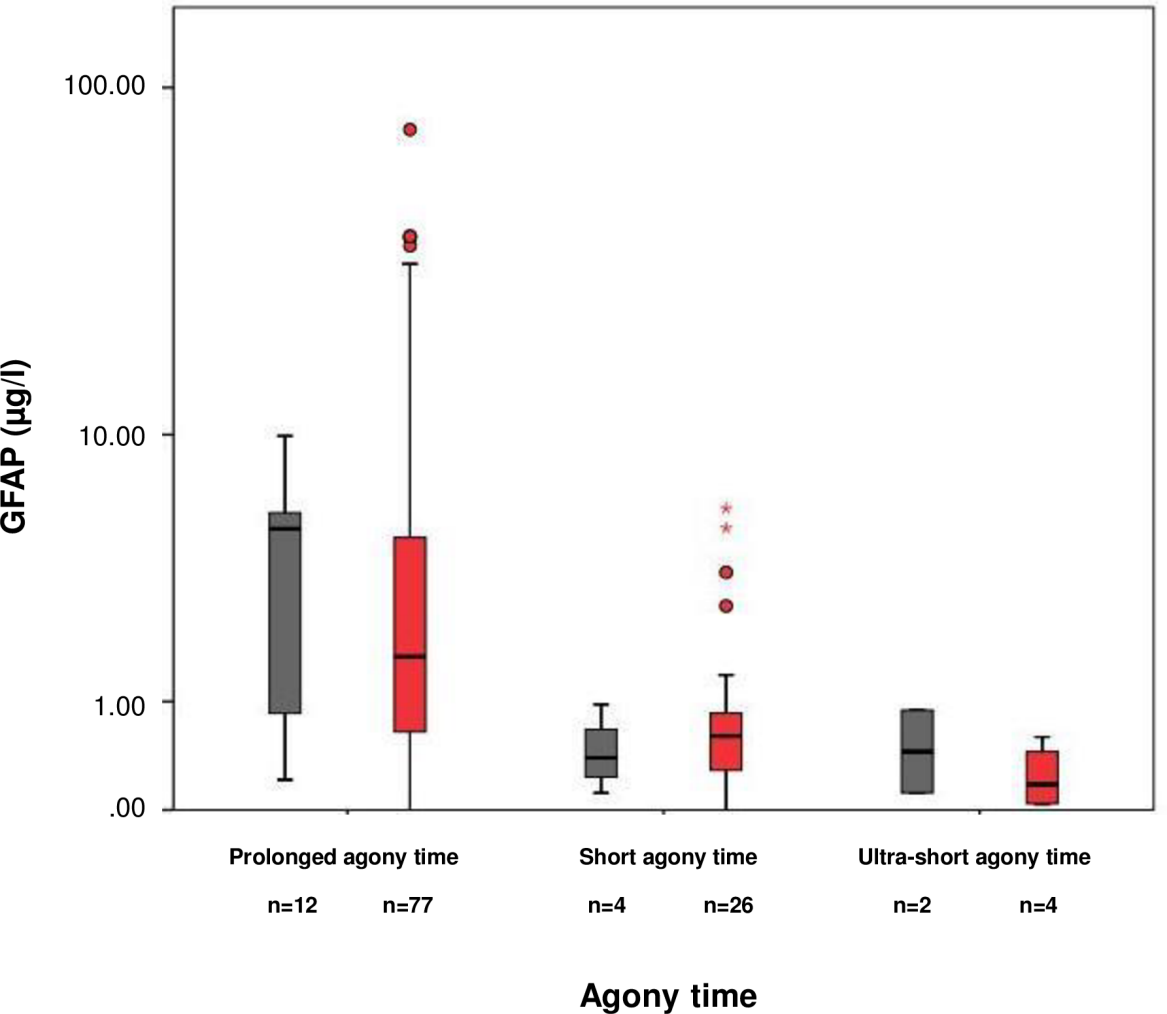
Post-mortem GFAP serum concentrations of the primary cerebral and the primary non-cerebral cause of death group stratified according to agony time. GFAP concentrations of the cerebral cause of death group (grey bars): ultra-short agony times (median 0.53 μg/l [minimum 0.12, maximum 0.89]; n = 2), short agony times (0.39 μg/l [IQR 0.65, 0.12, 0.96]; n = 4), and prolonged agony times (5.02 μg/l [5.26, 0.21, 9.91], n = 12; Kruskal Wallis p = 0.003; post-hoc ultra-short versus prolonged agony times p = 0.051, short versus prolonged agony p = 0.043). GFAP concentrations of the non-cerebral cause of death group (red bars): ultra-short agony times (median 0.19 μg/l [IQR 0.49 minimum 0.03, maximum 0.59]; n = 4), short agony times (0.60 μg/l [0.57, 0.00, 5.87]; n = 26), and prolonged agony times (1.66 μg/l [4.21, 0.28, 76.25], n = 77; Kruskal Wallis p<0.001; post-hoc ultra-short versus prolonged agony times p = 0.009, short versus prolonged agony p<0.001).

**Fig 3:**
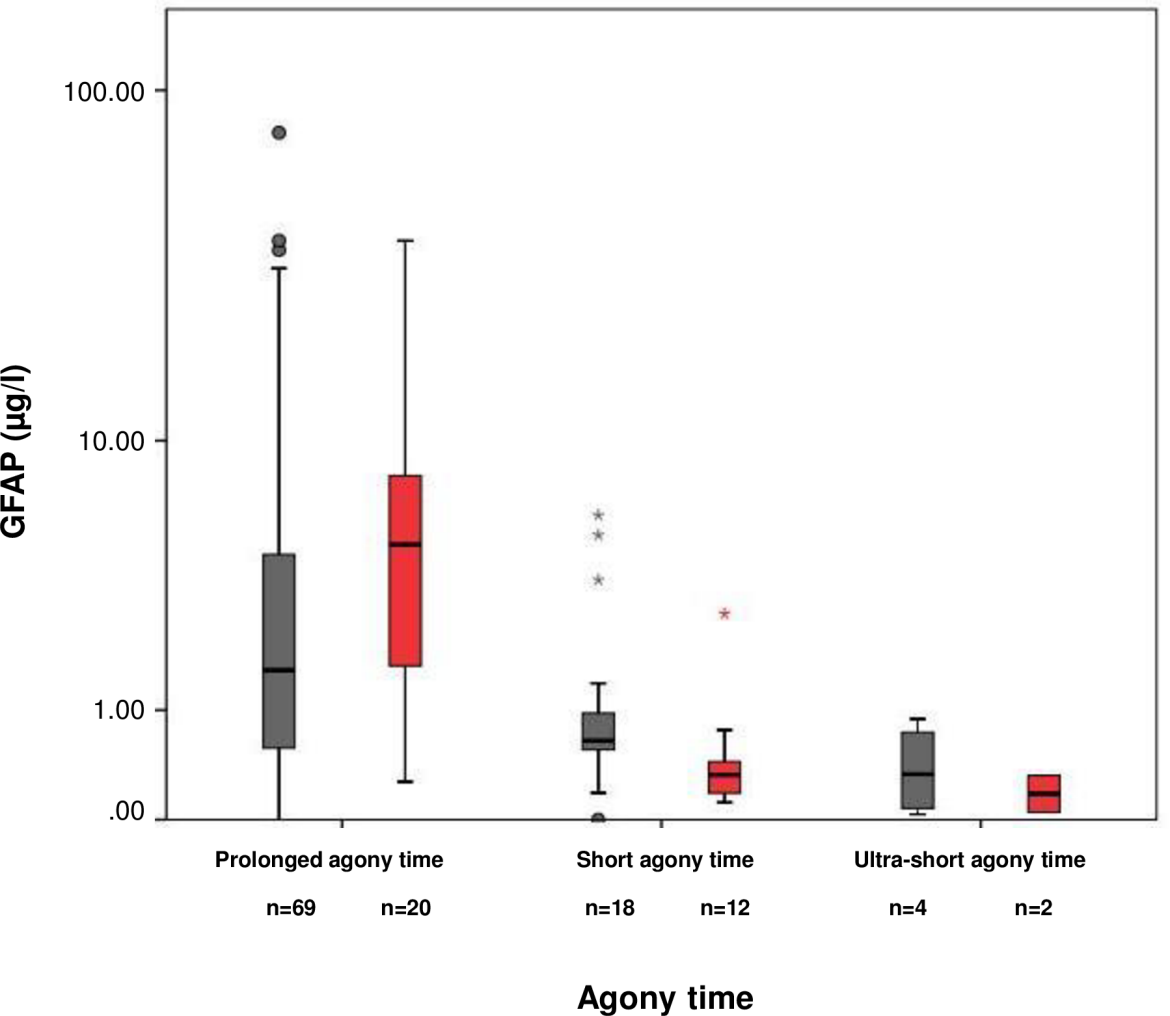
Post-mortem GFAP serum concentrations of the *brain injury group. Post-mortem GFAP serum concentrations of the *brain injury group (including all cases with a primary cerebral cause of death, all cases having macroscopic changes in brain integrity [such as cerebral edema and cerebral hemorrhage], and all cases in which the brain was exposed to hypoxia or ischemia [i.e., respiratory cause of death, exsanguination, multi-organ-failure, resuscitation]) and the control group (without brain injury) stratified according to agony time. GFAP concentrations of the brain injury group (grey bars): ultra-short agony times (median 0.35μg/l [IQR 0.76, minimum 0.03, maximum 0.83]; n = 4), short agony times (0.64μg/l [0.51, 0.10, 5.87]; n = 18), and prolonged agony times (1.58μg/l [3.91, 0.21, 76.25], n = 69; Kruskal Wallis p = 0,018; post-hoc ultra-short versus prolonged agony times p = 0.032, short versus prolonged agony times p = 0.041) GFAP concentrations of the control group (without brain injury; red bars): ultra-short agony times (median 0.19μg/l [IQR 0.27, minimum 0.05, maximum 0.32]; n = 2, p = 0.643), short agony times (0.33μg/l [0.30, 0.12, 2.68]; n = 12), and prolonged agony times (4.71μg/l [7.33, 0.27, 38.00], n = 20; Kruskal Wallis p<0.001; post-hoc ultra-short versus prolonged agony times p = 0.040, short versus prolonged agony times p<0.001).

We examined the influence of agony time on GFAP levels (Fig 1). N = 6 cases were grouped as having ultra-short agony time, n = 30 as having short agony time, and n = 93 as having prolonged agony time. GFAP concentrations were found low in cases with ultra-short agony times (0.21 μg/l [0.12, 0.03, 0.89]; n = 6), elevated in cases with short agony times (0.58 μg/l [0.58, 0.00, 5.87]; n = 30), and markedly increased in cases with prolonged agony times (1.76 μg/l [4.68, 0.00, 76.25], n = 89; Kruskal Wallis p<0,001; post-hoc ultra-short versus prolonged agony times p = 0,002(MWU), short versus prolonged agony times p<0,001(MWU)). We did not observe a difference in GFAP levels between cases with a cerebral cause of death and non-cerebral cause in any of the three agony time strata (Fig 2). This was also true for the brain injury group (including the cases with macroscopic and hypoxic / ischemic brain damage, see above; Fig 3).

## Discussion

Our prospective study revealed that the post-mortem serum concentration of the brain-specific astroglial protein GFAP is not an indicator of a neurological cause of death. Rather, we found that post-mortem GFAP increase in parallel with longer agony times. GFAP may provide insights into pathophysiological processes in the peri-mortal phase.

Our study did not provide evidence for differences in GFAP blood concentrations between individuals with a cerebral and a non-cerebral cause of death. Even when the cerebral cause of death group was enriched with the cases having macroscopic brain damages and the cases in which the brain was exposed to hypoxia or ischemia, a difference in GFAP serum concentrations was not observed. GFAP is released in case of intracerebral hemorrhage or massive traumatic brain injury (e.g., resulting from shooting) leading to death. However, it appears that other circumstances in the peri-mortal phase also affect GFAP levels, which then mask any additional release from the brain. Perimortal processes are likely to affect blood brain barrier integrity and disintegrate astroglial cells (regardless of any additional brain injury or additional hypoxic or ischemic stress). This may explain the slightly higher GFAP values as compared to healthy (living) controls. Indeed, it is conceivable that patients who undergo resuscitation or who had sustained minimal circulation show increased GFAP values as a consequence of hypoxic brain damage and blood-brain barrier dysfunction.[12] The release of astroglial proteins was previously found to correlate with the extent of hypoxic brain damage.[12] Our finding that GFAP levels are associated with the duration of the agony time supports this assumption. Interestingly, individuals with very short agony times showed the lowest GFAP levels. Here, a sudden circulation arrest can be assumed, leaving no time for the protein to be released out of hypoxic cells into the CSF and the blood stream. Future studies may analyze the release of astroglial proteins in the perimortal phase in more detail.

GFAP measurements in post-mortem serum samples have never been reported so far. GFAP is a very robust protein that has been shown to be stable in whole blood for days. [6]It can be detected in post mortem brain specimens, both in human and rodents.[15, 16, 19, 20] GFAP can be reliably determined if taken via a central venous line (e.g. in case of intensive care patients).[6, 21, 22] Furthermore, it can be reliably measured by standard ELISA techniques.[23] Although a correlation between the post mortem interval and level of hemolysis was found, there was no correlation between GFAP levels and the level of hemolysis. Thus we do not believe that hemolysis has critically interfered with our measurements. On the other side, effects of putrefaction and decay on brain biomarkers are not well understood, and there is no clear “time window” for valid GFAP determination after death. The fact that we were able to obtain GFAP levels in a wide range (0–76 μg/l) points to a successful determination of the protein. Indeed, other studies revealed GFAP levels in a comparable level.[8, 9, 10, 24].

Strength of this study was that the autopsy itself as well as blood sampling and GFAP measurements were performed in a highly standardized matter and thus allowed good comparison of inter-individual data. As a shortcoming, although we tried to account for technical difficulties resulting from the post-mortem blood sample itself (including longer time intervals between death and blood sampling), an interference of biochemical factors on the validity of GFAP analysis cannot be entirely ruled out. We decided to take heart blood out of the right cardiac chamber. Thus we were unable to compare blood specimens from different compartments. In retrospect, the primary analysis was hampered by a certain overlap between cases with a primary cerebral cause of death and cases having a primary non-cerebral cause of death in terms of (primary or secondary) structural brain damage in autopsy. We tried to minimize this drawback by enriching our cerebral cause of death group with all cases having macroscopic brain damage and all cases that were exposed to hypoxia and ischemia.

We renounced the analysis of astroglial proteins in CSF. Any severe brain injury will massively affect protein concentrations in the CSF. The collection of CSF was therefore not part of our prospective investigational protocol. Validation by histopathological analysis of brain samples was not performed in our study. However, in view of our findings, immunohistochemical analysis are urgently needed to study astrocytic injury and blood brain barrier dysfunction in post mortem brain tissue in more detail, in order to compare the release kinetics of GFAP under hypoxic (in terms of ‘primary non cerebral cause of death’) and traumatic (in terms of ‘primary cerebral cause of death’) conditions.

The primary intention of this study was to identify a biomarker that indicates a cerebral cause of death. A search for GFAP in a blood spot found at a crime scene where a body or corpse is missing could, therefore, help clarify whether a severe primary cerebral injury existed.[25] However, according to the results of our study, pathophysiological events in the peri-mortal phase also provoke GFAP release, thus making differentiation impossible. Nevertheless determination of the agonal interval is still part of active research in the current forensic practice. Up to now no clear serological marker has been encountered to specify length of agony time. Post mortem serological catecholamine levels and immunohistochemical analysis of ubiquitin in the locus coeruleus have shown promising results. [17, 18] The astroglial protein S100B and the neuronspecific enolase (NSE) have recently been investigated with regard to traumatic brain injury. [26] Post mortem serum levels of both markers were not elevated in trauma cases compared to controls, but CSF levels of both biomarkers correlated with length of survival time after trauma. [26] Future studies may now be performed to assess the connection between astroglial proteins (including GFAP and S100B), NSE and agony time in more detail.[27].
